# Duodenum Inversum in a Female: A Rare Clinical Entity Presenting With Right Hypochondrial Pain

**DOI:** 10.7759/cureus.68512

**Published:** 2024-09-03

**Authors:** Aneeqa Qureshi, Huzafa Ali, Jeevan Gyawali, Aisha Tariq, Maheen Kamran

**Affiliations:** 1 Radiology, Aga Khan University Hospital, Karachi, PAK; 2 Internal Medicine, Combined Military Hospital (CMH) Multan Institute of Medical Sciences, Multan, PAK; 3 Internal Medicine, Chirayu National Hospital and Medical Institute, Kathmandu, NPL; 4 Internal Medicine, Karachi Medical and Dental College, Karachi, PAK; 5 Internal Medicine, Quaid-e-Azam Medical College, Bahawalpur, PAK

**Keywords:** congenital malformation, diagnostic imaging, duodenum inversum, gastrointestinal anomalies, right hypochondrial pain

## Abstract

Duodenum inversum is a rare congenital anomaly in which the proximal duodenum travels posteriorly and superiorly before crossing the midline, often presenting asymptomatically. Clinical features can include epigastric pain, nausea, and abdominal distension. This case report describes a 35-year-old female who presented with right hypochondrial pain. A CT scan revealed that the third part of the duodenum followed an upward and vertical course, crossing the midline at a higher level than usual, thus confirming the diagnosis of duodenum inversum. Despite the absence of obstruction, conservative medical management was employed to address the symptoms. This case highlights the importance of considering duodenum inversum in the differential diagnosis of right hypochondrial pain and underscores the value of modern imaging techniques in accurate diagnosis. Awareness of this rare condition can facilitate timely and effective management.

## Introduction

Duodenum inversum, also known as duodenum reflexum or inverted duodenum, is a rare congenital malformation in which the third segment of the duodenum follows a superoposterior course before crossing the midline above the pancreas instead of proceeding leftward to the ligament of Treitz [1]. It is more commonly observed in males, with a male-to-female ratio of approximately 4:1. Since 1950, fewer than 20 cases have been documented, with an incidence rate of 0.07% [2].

The condition is believed to result from a persistent dorsal mesentery within a movable duodenum [3]. It may present similarly to superior mesenteric artery syndrome due to a medial linear extrinsic impression on the duodenum or compression against the psoas muscle, particularly in the context of sudden weight loss. Although duodenum inversum is often asymptomatic, it can present with obstructive symptoms such as epigastric discomfort, nausea, vomiting, and abdominal distention. The condition may increase susceptibility to pancreatitis and peptic ulcers due to stasis of ingested materials in the duodenum [4]. In adults, it is occasionally associated with other gastrointestinal anomalies, such as malrotation, annular pancreas, and pancreatic divisum, but these findings are usually incidental and do not require surgical intervention unless obstruction is evident [3]. Patients may also experience pancreatitis or gallbladder disease due to partial or functional obstruction of the biliary system.

Diagnosis typically involves radiological evaluations for patients presenting with abdominal pain, but the condition may also be discovered incidentally during surgery or autopsy. Laparoscopy and hypotonic duodenography can be useful diagnostic tools. Fluoroscopic upper gastrointestinal examinations are critical for accurate diagnosis. For mild cases, conservative management may be sufficient; however, surgical intervention remains the gold standard for severe cases, particularly when obstruction is apparent [5]. This case is noteworthy due to the rarity of the congenital anomaly, particularly in females, and highlights the need for ongoing research into its etiology and management.

## Case presentation

A 35-year-old female with a known history of chronic bronchitis presented to the outpatient department with persistent pain localized to the right hypochondrium, which had lasted for the past month. The pain, described as sharp, was exacerbated by actions such as coughing, sneezing, and bending forward. She experienced some relief from the pain by taking analgesics and applying pressure to the affected area. Her surgical history included an appendectomy during childhood and three cesarean deliveries, the most recent of which occurred two years ago. On clinical examination, her abdomen was soft and non-tender.

The preliminary diagnostic workup included liver function tests, which returned normal results. However, blood tests revealed normocytic hypochromic anemia, with mean corpuscular hemoglobin concentration at 30.1 g/dl (normal: 31-34 g/dl) and mean corpuscular hemoglobin at 19.1 pg (normal: 27-32 pg). A posteroanterior chest X-ray did not show any significant abnormalities. An abdominal ultrasound indicated hepatomegaly, with liver size measuring 15.6 cm and no intra- or extrahepatic ductal dilatation or calcification.

**Figure 1:**
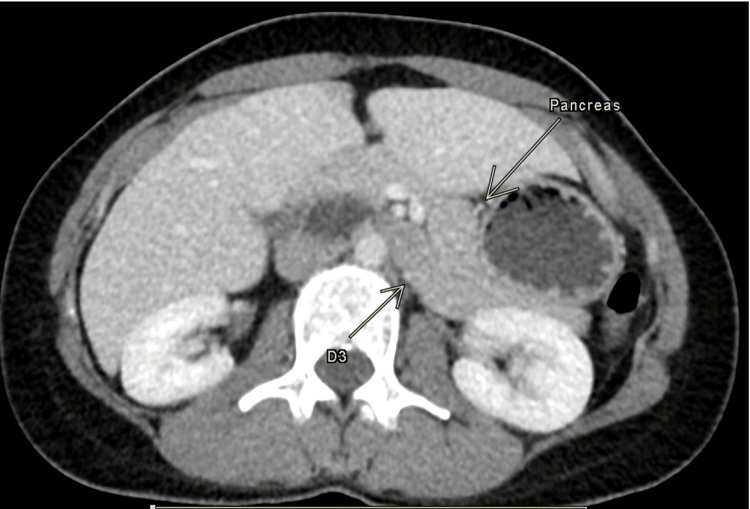
Post-contrast CT of the abdomen showing the third part of the duodenum (D3) at the level of the pancreatic body

**Figure 2:**
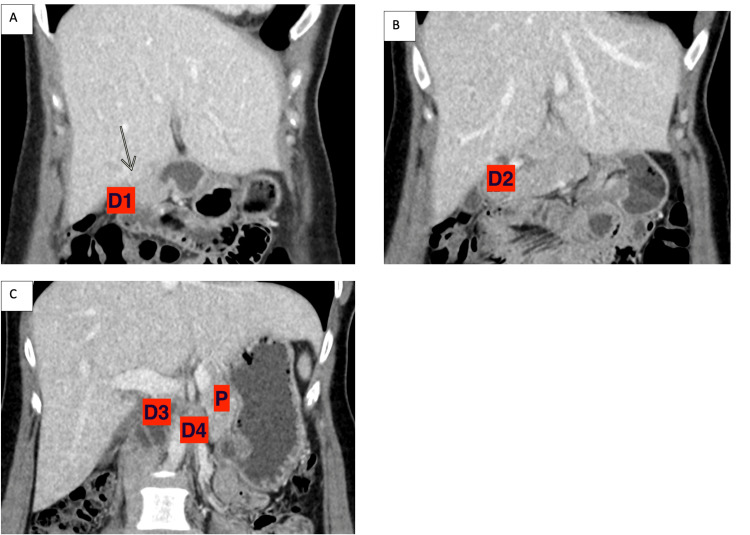
Contrast-enhanced CT of the abdomen showing the third part of the duodenum positioned higher relative to the second part and crossing the midline at the level of the pancreatic body (A) At the level of the first part of the duodenum (D1). (B) At the level of the second part of the duodenum (D2). (C) At the levels of the third part (D3) and fourth part (D4) of the duodenum.

A subsequent CT scan of the abdomen with intravenous contrast revealed that the third portion of the duodenum turned to the right and followed an upward and vertical course, curving to the left and crossing the midline above the pancreas (Figure 1). Instead of turning to the left, it crossed the midline and formed the duodenojejunal junction (Figure 2). There were no signs of gut obstruction or malrotation detected.

She was treated with flurbiprofen, esomeprazole magnesium, and the combination drug paracetamol with tramadol. Her symptoms improved throughout her hospital stay, and she was discharged in good health. She had no symptoms upon follow-up.

## Discussion

Duodenum inversum is an exceptionally rare congenital anomaly, with only about 20 cases reported in the literature to date. This condition often presents with symptoms similar to those of intestinal malrotation, making the identification of the typical duodenojejunal junction crucial for differentiating between various types of malrotation [6,7].

The presented case highlights the rarity of this congenital anomaly, with only a few patients affected. While duodenum inversum generally does not present with symptoms, it may occasionally cause right-sided hypochondrial pain. This pain is often attributed to other conditions such as cholelithiasis, cholecystitis, hepatitis, or other liver and gallbladder diseases like cholangitis, hepatoma, and cirrhosis. Therefore, considering duodenum inversum in the differential diagnosis for right hypochondrial pain is important, especially when common causes have been ruled out.

Duodenum inversum is more prevalent in males, with a reported ratio of 4:1. The case presented here, involving a female in her mid-30s, is particularly noteworthy [8]. The patient exhibited only right hypochondrial pain, which led to further diagnostic investigation. A CT scan of the abdomen confirmed the diagnosis, providing a new perspective on this case, as duodenum inversum is typically diagnosed through an upper gastrointestinal series or laparoscopy [9]. The subtle fluoroscopic appearance of malrotation can sometimes mimic other physiological or pathological conditions, creating a diagnostic challenge. Accurate performance of upper GI studies is essential, and radiologists must be well versed in normal variations of duodenal anatomy [10].

Feldman and Morrison classified duodenum inversum into four types: Type 1 (complete inversion with no visible duodenal curve), Type 2 (identifiable duodenal curve), Type 3 (duodenal curve with marked redundancy), and Type 4 (associated with malrotation) [11]. This case corresponds to Type 2, as the contrast-enhanced CT scan shows the third part of the duodenum positioned higher relative to the second part, crossing the midline at the level of the pancreatic body, thus displaying an identifiable duodenal curve.

Despite these anatomical variations, patients often have a normal position of the duodenojejunal junction, which can lead to missed diagnoses due to the asymptomatic nature of the condition. This underscores the importance of advanced imaging modalities for accurate diagnosis and highlights the need for clinical awareness to avoid overlooking potential cases. Complications of duodenum inversum may include duodenitis, acute pancreatitis, peptic ulcer disease, functional biliary obstruction, and duodenal volvulus. Symptomatic patients without evidence of obstruction are generally managed conservatively, as was the case with our patient, with favorable outcomes. If obstruction develops or conservative management fails, surgical intervention may be necessary [12].

Limitations of this case include its singular nature, which limits the generalizability of the findings. Conclusions drawn from a single case may not be broadly applicable. Additionally, long-term follow-up is lacking, leaving a gap in understanding the overall prognosis and outcomes of such pathologies. Finally, the inherent selection bias in reporting unique cases may lead to an overemphasis on the clinical significance of rare events.

## Conclusions

This case report highlights the rarity of duodenum inversum, adding a unique perspective with its presentation in a female patient. While duodenum inversum is typically asymptomatic, it can occasionally present with unusual symptoms such as right hypochondrial pain, necessitating a high index of suspicion and thorough diagnostic evaluation. This case underscores the importance of CT scans and upper gastrointestinal series in accurately diagnosing duodenum inversum and differentiating it from other gastrointestinal anomalies. The Type 2 duodenum inversum observed in this patient was characterized by the third part of the duodenum being positioned anomalously relative to the second part, crossing the midline at the level of the pancreatic body. This report contributes to the limited literature on duodenum inversum and emphasizes the need for continued awareness and understanding to ensure prompt and accurate diagnosis and management of this rare condition.
